# Phytonutritional Content and Aroma Profile Changes During Postharvest Storage of Edible Flowers

**DOI:** 10.3389/fpls.2020.590968

**Published:** 2020-11-27

**Authors:** Ilaria Marchioni, Laura Pistelli, Benedetta Ferri, Andrea Copetta, Barbara Ruffoni, Luisa Pistelli, Basma Najar

**Affiliations:** ^1^Department of Agriculture, Food and Environment (DAFE), University of Pisa, Pisa, Italy; ^2^Interdepartmental Research Center NUTRAFOOD “Nutraceuticals and Food for Health”, University of Pisa, Pisa, Italy; ^3^Department of Pharmacy, University of Pisa, Pisa, Italy; ^4^Research Centre for Vegetable and Ornamental Crops (CREA), Sanremo, Italy

**Keywords:** shelf-life, functional food, bioactive compounds, volatile compounds, Asteraceae, Geraniaceae, Lamiaceae

## Abstract

Edible flowers are niche horticultural products, routinely used as cooking ingredients in the food industry. Currently, new species are required with the aim of enlarging the number of species with a long shelf-life, healthy nutraceutical compounds, and new fragrance and tastes. *Ageratum houstonianum* Mill, *Tagetes lemmonii* A. Gray, *Salvia dorisiana* Standl, and *Pelargonium odoratissimum* (L.) L’Hér “Lemon” were selected for their different morphological characteristics and color. Fresh flowers were analyzed to characterize their phytonutritional content and aroma profile. Postharvest was determined up to 6 days of cold storage at 4°C in transparent polypropylene boxes. Visual quality and cellular membrane damage were observed. The relative content of different antioxidant constituents (e.g., polyphenols, flavonoids, anthocyanins, ascorbic acid), nutritional compounds (soluble sugars, crude proteins), the antioxidant scavenging activity, and the volatile profile were determined and correlated to the quality of shelf-life of the different species. The yellow *T. lemmonii* freshly picked flowers showed the highest ascorbic acid and flavonoids content, which was maintained during the cold storage, as well as the best visual quality. Limited changes in metabolites were detected in the light blue *A. houstonianum* during postharvest, although the visual quality is severely compromised. Magenta *S. dorisiana* and light pink *P. odoratissimum* showed similar changes in antioxidant constituents during cold storage. For the first time, the volatile compounds have been identified in the four species. Sesquiterpene hydrocarbons are the main class in fresh flowers of *A. houstonianum*, *S. dorisiana*, and *P. odoratissimum*, while monoterpene hydrocarbons are abundant in *T. lemmonii.* The cold storage influenced mainly *P. odoratissimum* and *S. dorisiana* flavor initially dominated by the increase in total monoterpenes at 6 days, reaching a relative content of 90%. Both *A. houstonianum* and *T. lemmonii* conserved the prevalence of the same class of constituents in all the analyzed conditions, even though the cold storage influenced the major compound abundance. On the basis of the results, *T. lemmonii* was the most interesting species with the longest shelf-life due to its phytonutritional and aromatic constituents. Results indicated the peculiar metabolic and physiological attitude of flowers species to cold storage.

## Introduction

Edible flowers (EFs) were traditionally used over the centuries in various part of the world as ingredient of sweet and savory dishes (Mlcek and Rop, 2011; Cunningham, 2015). Nowadays, the crops with EFs are increasing because of their aromatic and healthy properties (Chen and Wei, 2017). Flowers are esthetically appreciated for their different colors (Kelley et al., 2001a, b, 2002) and are characterized by sweet, sour, and spicy flavors and aroma (Benvenuti et al., 2016; Fernandes et al., 2018b; Simoni et al., 2018). From the nutritional point of view, EFs are safe because of the organic cultivation; they are healthy and rich in beneficial substances, including polyphenols (Lu et al., 2016; Fernandes et al., 2017), essential amino acids (Sotelo et al., 2007), minerals (Rop et al., 2012; Tokalıoğlu, 2012; Grzeszczuk et al., 2018; Drava et al., 2020), polyunsaturated fatty acids (reviewed in Fernandes et al., 2018b), ascorbic acid (Grzeszczuk et al., 2016), vitamin E (Miguel et al., 2016; Pires et al., 2017; Fernandes et al., 2018b), and xantophylls (Fernandes et al., 2018b). Most of these molecules have antioxidant, antimicrobial, and anti-inflammatory properties (Lu et al., 2016).

To ensure a quality product, EF sale and storage have to cope with the weakness of perishability. Visually perfect flowers must be picked with care, packed properly in order to protect them from any mechanical damage, and stored at proper temperature until consumption (Fernandes et al., 2020). In these conditions, shelf-life generally is from 2 to 10 days (Fernandes et al., 2019b, 2020) until the onset of the senescence process. Loss of color, wilting, dehydration, fast browning, and petal abscission (Kelley et al., 2001b; Rani and Singh, 2014; Landi et al., 2017) are often accompanied by membrane damages and decline in bioactive compounds (Kou et al., 2012; Landi et al., 2017; Demasi et al., 2020).

Postharvest technologies are common methods used in ornamental and aromatic flowers to extend the shelf-life because of their high economical value (Fernandes et al., 2020). Usually, the flowers are maintained at low temperatures (generally around 5–10°C) to delay the senescence process (Menegaes et al., 2019), and the high presence of antioxidant molecules counteracts the production of free radicals (Mlcek and Rop, 2011; Cavaiuolo et al., 2013). The scavenger activity is performed largely by several bioactive compounds as polyphenols, carotenoids, and ascorbic acid, which are the most relevant in flowers (Mlcek and Rop, 2011; Cavaiuolo et al., 2013). Thus, high levels of antioxidants during the postharvest storage could be useful to guarantee a long shelf-life, as reported in different EFs, which maintained their marketability, polyphenols and anthocyanins content up to 8 postharvest days (Landi et al., 2017). Nevertheless, during cold storage, ascorbic acid content follows different trends depending on the plant species: this metabolite decreased progressively in *Cucurbita pepo* (Aquino-Bolaños et al., 2013), *Tulbaghia cominsii*, and *Tropaeolum majus* (Landi et al., 2017); a significant increase in ascorbic acid was observed in *Acmella oleracea* after 2 postharvest days (Landi et al., 2017), and no changes were observed in two different *Salvia* species (Landi et al., 2015, 2017).

Fragrance is another important characteristic of EF to be remained pleasant until consumption. To date, very few studies have been carried out to identify their aroma (Fernandes et al., 2019a; Najar et al., 2019; Marchioni et al., 2020), both in fresh flowers and during their conservation (Koksall et al., 2015; Schmitt et al., 2018).

The increased interest and EU research programs related to EF (Fernandes et al., 2020) offer the opportunity to investigate new species to be used as functional food, with the aim of increasing the choice in the market. *Ageratum houstonianum* Mill. (Asteraceae), also known as floss flower, is an annual plant native to Central America (Benvenuti et al., 2016). It is a popular garden plant cultivated in Europe since 1800 (Johnson, 1971). The inflorescences are dense corymbs composed of small light blue, lilac, or lavender flowers, the taste of which is similar to carrot (Benvenuti et al., 2016).

*Tagetes lemmonii* A. Gray (Asteraceae), or Lemmon’s marigold, is a perennial herbaceous plant, which is native to North America (Ma et al., 2018). Both ray and disk florets are edible, characterized by strong lemon taste and fragrance. Other flowers belonging to the same genus are edible and currently consumed, such as *Tagetes erecta* L. (African marigold) and *Tagetes tenuifolia* Cav. (signet marigold) (Newman and O’Connor, 2009).

*Salvia dorisiana* Standl (Lamiaceae), also called fruit sage or peach sage, is an herbaceous plant indigenous to Honduras and probably to other parts of Central America (Halim and Collins, 1975). The common plant name came from the intense fruity aroma emitted by leaves if rubbed (Cervelli, 2011). The same flavor can be perceived by eating its magenta flowers; both flowers and leaves are edible, and they are appreciated in sweet preparations (Cervelli, 2011).

*Pelargonium odoratissimum* (L.) L’Hér “Lemon” (Geraniaceae) is a variety of the more common species *P. odoratissimum.* The variety has petals with a surprising lemon taste, and there are no reports on its use. It is a perennial aromatic shrub indigenous to South Africa, widely cultivated as ornamental (Christodoulakis et al., 2013). The essential oils (EOs) of the common species can be used in perfumery and aromatherapy (Andrade et al., 2011; Christodoulakis et al., 2013), and their antimicrobial activity was well documented (Lis-Balchin et al., 1998; Andrade et al., 2011).

In this study, four different EFs (*A. houstonianum*, *T. lemmonii*, *S. dorisiana*, and *P. odoratissimum*) differing in color, aroma, and morphology were investigated to evaluate their nutraceutical properties as antioxidant constituents and aroma profile. Moreover, the postharvest treatment at 4°C for 2 and 6 days was performed in order to explain the role of these antioxidant compounds to clarify the storage attitude at cold temperature of these species.

## Materials and Methods

### Plant Material and Cold Storage

The four species *A. houstonianum* Mill., *T. lemmonii* A. Gray, *S. dorisiana* Standl., and *P. odoratissimum* (L.) L’Her “Lemon” were grown in the greenhouse covered with insect proof net, located at CREA Research Centre for Vegetable and Ornamental Crops (CREA, Sanremo, IM, Italy, GPS: 43.816887, 7.758900). Seeds of *A. houstonianum* were sown in spring 2018 and cultured in pots (14 cm of diameter, 0.5 L). *P. odoratissimum* “Lemon,” *S. dorisiana*, and *T. lemmonii* plants derived from softwood cuttings prepared in September, April, and May 2018, respectively, were cultured in pots (30 cm of diameter; 9 L). Substrate composition, fertilization treatment, and frequency and type of irrigation were described in Marchioni et al. (2020). The plants were grown organically, as reported in Najar et al. (2019). Fresh flowers of the four species were harvested in their full maturity in February 2019. After collection (around 20 g), each species was equally divided into three different pools representing three experimental points: fresh flowers, day 0 (T0), postharvest treatment for 2 days (T2) and 6 days (T6). The postharvest storage was carried out using disposable transparent polypropylene boxes (120 × 85 × 80 mm, equal to 800 ml of volume) with hermetically sealed lid and stored at 4°C in a refrigerator in dark condition. T0 samples were immediately evaluated for the visual quality (as described in *Flowers Visual Quality Rating*), quickly frozen in liquid nitrogen, and stored at −80°C until biochemical analyses. Flower spontaneous volatile emission was detected quickly at the end of each experimental point. Experiments were repeated in triplicate, maintaining the same experimental conditions to ensure the validity of the data.

### Weight Loss

Initial flower weight was measured by an analytical balance (Ohaus^®^ analytical Standard Series^TM^ Model AS60S, Ohaus Corporation, Florham Park, NJ, United States) before cold storage (M_0_). At the end of each experiment, the flowers were weighted again (M). Weight loss was calculated according to Fernandes et al. (2018a), following this equation:

$$\mathrm{WL}=\frac{{\text{M}}_{\text{o}}-\text{M}}{{\text{M}}_{\text{o}}}\times 100$$

### Flowers Visual Quality Rating

Freshly picked (T0) and cold-stored flowers (T2 and T6) were rated visually using a 4-point scale, as reported in Table 1. Scale points were defined properly for each species under evaluation, based on their specific attitude to cold storage: from 1 (severe loss of quality) to 4 (flawless flowers). Only flowers rated as 4 were carefully further used for the experimental design. For each experimental point, scores were calculated as average of the visual evaluation performed on 15 different and representative flowers.

**TABLE 1:** Postharvest visual quality ratings (4 = highest quality; 1 = lowest quality).

Species	Rating			

	1	2	3	4
*Ageratum houstonianum*	Severe loss of florets turgor, browning of tubular florets and their detachment if handled	Moderate loss of florets turgor, limited color changes	Very limited loss of florets turgor, no visible color changes	No defects
*Tagetes lemmonii*	Substantial inflorescence color changes (yellow turns in darker hue), most of ray florets are curled	Slight browning of the inflorescence, tips of ray florets moderately curled	Very limited color changes, no visible loss of turgor	No defects
*Salvia dorisiana*	Browning of the corolla tube, homogeneous corolla discoloration	Slight browning of the corolla tube, visible discoloration in the apical part of the corolla	Limited color changes, no visible loss of turgor	No defects
*Pelargonium odoratissimum* “Lemon”	Petals wilted with dark margins, detachment of most of the petals	Petals curled moderately, limited color changes, detachment of few petals if handled	Petals curled slightly, very limited color changes of pink striations	No defects

### Lipid Peroxidation (TBARS Assay)

Lipid peroxidation was determined by the thiobarbituric acid reactive substances (TBARS) method (Hodges et al., 1999) after extraction of 200 mg of fresh flowers with 1 ml of 0.1% (w/v) trichloroacetic acid (TCA) solution. The malondialdehyde (MDA) concentration was determined at 532 nm. Samples were read also at 440 and 660 nm to account for interferences. TBARS content was expressed as nanomoles of MDA equivalents (MDAe) per gram of fresh weight.

### Total Phenolic and Flavonoid Content

Two hundred milligrams of fresh flowers was extracted with 2 ml of 70% methanol (v/v) as described in Najar et al. (2019). The extracts were also used for the determination of the antioxidant activity and the total flavonoid content (TFC). Total phenolic content (TPC) was performed by Folin–Ciocalteu method (Singleton and Rossi, 1965) and was expressed as milligrams of (+)-catechin equivalents (CE) per gram of fresh weight. The TFC was determined as reported in Kim et al. (2003) with some modifications (Caser et al., 2018). The absorbance was read at 510 nm, and the TFC was expressed as milligrams of (+)-catechin equivalents (CE) per gram of fresh weight.

### Total Anthocyanin Content

Anthocyanins were extracted from 100 mg of fresh flower with 0.5 ml of 99/1% ethanol/HCl as described in Cheng and Breen (1991). The sample was read at 535 nm, and the total anthocyanin content was expressed as milligrams of malvin chloride equivalents (MEs) per gram of fresh weight.

### DPPH Radical-Scavenging Activity Assay

The antioxidant activity was determined using the 2,2-diphenyl-1-picrylhydrazyl radical (DPPH) scavenging method (Brand-Williams et al., 1995). This effect (%) was calculated as $\left[\left(\frac{\mathrm{Abs0}-\mathrm{Abs1}}{\mathrm{Abs0}}\right)\times 100\right]$, where Abs0 is the absorbance of the control and Abs1 is the absorbance of the sample. Results were reported as IC_50_ (mg/ml), i.e., the milligrams of flowers required to obtain 50% of antioxidant activities (Molyneux, 2004).

### Total Ascorbic Acid Determination

Total ascorbic acid (ASA_*TOT*_) was quantified according to the method of Kampfenkel et al. (1995). Fresh samples (200 mg) were extracted in 2 ml of 6% (w/v) TCA solution as described by Degl’innocenti et al. (2005). The data were reported as milligrams of ASA_*TOT*_ per gram of fresh weight.

### Sucrose, D-Fructose, and D-Glucose Quantification

Soluble sugars extraction was performed following the method described in Tobias et al. (1992) with some modification. In brief, 1.2 ml of 5.5% (v/v) HClO_4_ was used to homogenize 100 mg of fresh flowers. The samples were used to quantify sucrose, D-fructose, and D-glucose concentrations. This determination was performed using a Sucrose/D-Fructose/D-Glucose Assay Kit (Megazyme International Ireland, Co., Wicklow, Ireland), following the manufacturer’s instructions. Samples of 0.010 ml were used for each determination. The results were expressed as milligrams of sucrose, D-fructose, or D-glucose per gram of fresh weight.

### Total Nitrogen Quantification and Crude Protein Content Estimation

Freeze-dried flowers (300 mg) were used to perform nitrogen content determination by Kjeldahl method, following the protocol described in Jones et al. (1991). Data were reported as percentage of crude protein content, obtained by multiplying the percentage of nitrogen by 6.25 as conversion factor (%N × 6.25) (Marchioni et al., 2020).

### Volatiles Analysis and GC-MS Analysis

Fresh flowers were transferred and stored in glass containers (ø 4.5 mm, h 80 mm) to avoid plastic volatile contamination. The spontaneous emission profiles of the studied flowers were analyzed using the method described in a previous study (Demasi et al., 2019). Volatiles were sampled from the flower of each species with a solid phase microextraction device (SPME, Supelco, Bellefonte, PA, United States) coated with polydimethylsiloxane (PDMS, 100 μm coating thickness, St. Louis, MO, United States). Then, they were injected into a Varian CP-3800 apparatus (Varian Inc., Palo Alto, Santa Clara, CA, United States) with a DB-5 capillary column (30 m × 0.25 μm i.d. film thickness 0.25 μm) coupled to a Varian Saturn 2000 (Varian Inc., Palo Alto, Santa Clara, CA, United States) ion-trap mass detector for the gas chromatography–mass spectrometry (GC-MS) analyses. The identification of constituents was based on the comparison of the retention times (Rt) with those of pure reference samples and their linear retention indices (LRIs) determined relatively to a series of *n*-alkanes. The mass spectra were compared with those listed in the known commercial libraries (NIST, 2014; Adams, 2007) and in a homemade mass spectra library built up from pure substances and components of known essential oils and MS literature data.

### Statistical Analysis

Weight loss, visual quality, and biochemical data were statistically analyzed using one-way ANOVA [using either Tukey’s honest significant difference (HSD) or the Games–Howell test according to the homogeneity of variance] (IBM SPSS software, IBM Corp. Released 2017. IBM SPSS Statistics for Windows, Version 25.0. Armonk, NY, United States) (Lee and Lee, 2018). The multivariate analysis was performed on volatile compounds present with a percentage ≥1%. The correlation matrix was used for the measurement of eigenvalues and eigenvectors in PCA where the plot was performed, selecting the two highest principal components (PCs). This analysis aimed at reducing the dimensionality of the multivariate data of the matrixes while preserving most of the variance (Choi et al., 2004). The two-way hierarchical cluster analysis (HCA) was performed using Ward’s method with squared Euclidian distances as a measure of similarity. These two analyses were conducted by the JMP software package 13.0.0 (SAS Institute, Cary, NC, United States). Linear correlation between antioxidant constituents and antioxidant scavenging activity (DPPH) was determined using Microsoft Excel^®^ 2013 (Microsoft Corporation, Redmond, WA, United States). The statistically significant differences induced by family and time of harvest on volatile organic compounds (VOCs) were assessed with two-way permutational multivariate analysis of variance (PERMANOVAs) with Euclidean distances, which were based on a distribution-free analysis of variance. This latter test was performed by Past 3 software, version 3.15.

## Results

### Weight Loss and Visual Quality During Cold Storage

Flowers were harvested (T0) and immediately stored at 4°C in plastic boxes to investigate their change in visual quality and biochemical profile that determine their shelf-life. During the 6 days of postharvest treatment (T6), the fresh weight of each species decreased significantly (Table 1). After 2 days of cold storage (T2), the decline of the fresh weight ranged between 3% in *T. lemmonii* and 11% in *A. houstonianum* and *P. odoratissimum*. At T6, the fresh weight decreased to 9.5% in *T. lemmonii* and 18% in *P. odoratissimum* “Lemon” (Table 2).

**TABLE 2:** Weight loss of *A. houstonianum*, *T. lemmonii*, *S. dorisiana*, and *P. odoratissimum* “Lemon,” at 0 (T0), 2 (T2), and 6 (T6) postharvest days (storage at 4°C).

	*A. houstonianum*	*T. lemmonii*	*S. dorisiana*	*P. odoratissimum* “Lemon”

Days	*Weight loss (%)*			
0	0^c^	0^c^	0^b^	0^c^
2	11.42 ± 0.42^b^	2.77 ± 0.07^b^	9.90 ± 1.11^a^	11.35 ± 0.39^b^
6	15.77 ± 1.54^a^	9.47 ± 1.09^a^	10.33 ± 0.69^a^	18.25 ± 1.20^a^

*Data are shown as mean ± SE; *n* = 3. Data are shown as mean ± SE; *n* = 3. Different letters indicate statistically significant differences (*P* < 0.05; Tukey’s HSD test) between experimental points of each species.*

**FIGURE 1:**
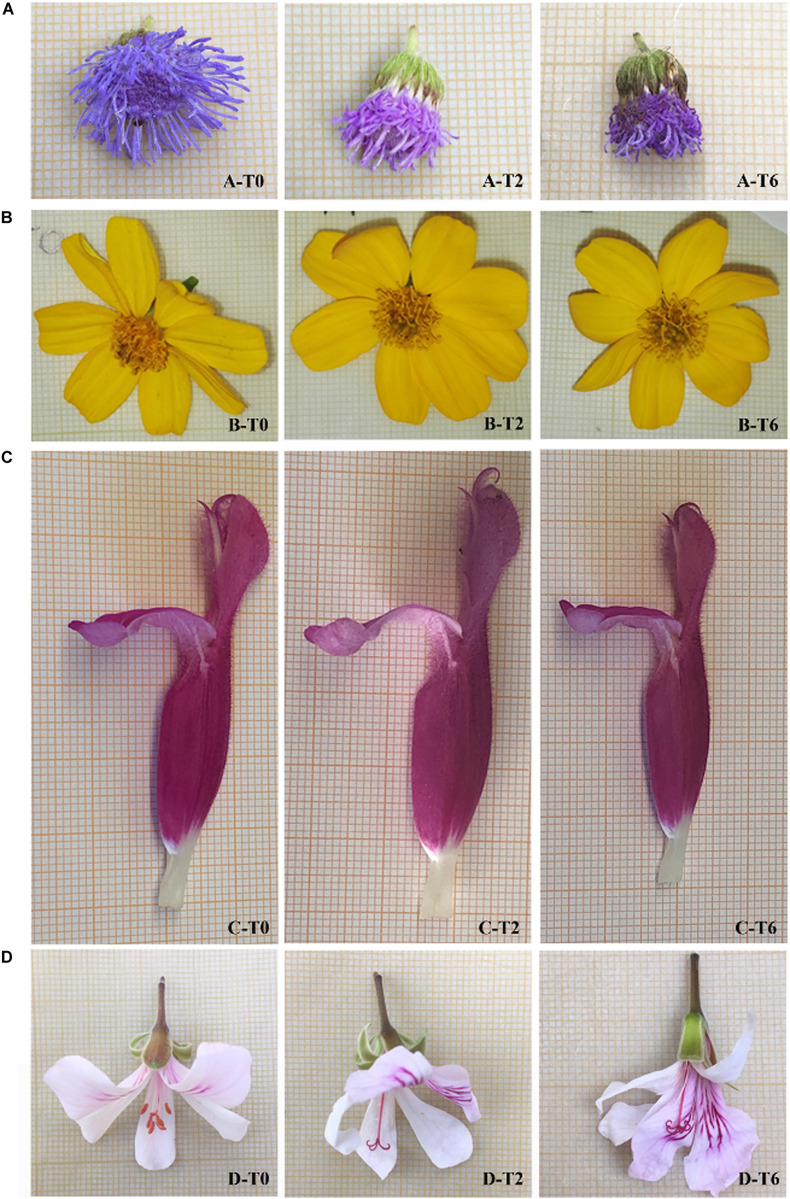
Visual appearance of four EFs (line A—*A. houstonianum*; line B—*T. lemmonii*; line C—*S. dorisiana*; line D—*P. odoratissimum*) after different times of cold storage (4°C): freshly picked flower (T0); after 2 days of cold storage (T2); and after 6 days of cold storage (T6). During the storage period (from T0 to T6), the appearance of the flowers changed according to the species: *A. houstonianum* showed evident browning of florets; *T. lemmonii* maintained the best visual quality until the end of the experiment; *S. dorisiana* flowers altered their pigmentation in the apical part of the corolla; petals of *P. odoratissimum* showed signs of loss of turgor, appeared wrinkled, and tended to detach from the corolla if handled.

In relation to weight loss, the aesthetic appearance changed (Figure 1 and Table 3). *A. houstonianum* flowers were the most negatively affected by cold storage, showing evident browning of florets and a likely loss of firmness (personal observation). The visual quality decreased at T2 of storage at 4°C (2.43 ± 0.31) and worsened further at T6 (1.50 ± 0.34) (Table 3). *T. lemmonii* maintained the best visual quality until the end of the experiment, showing only limited chromatic changes; indeed, at T2 and T6, the score of visual quality was statistically equal to that of the freshly picked flowers. *S. dorisiana* flowers altered their pigmentation in the apical part of the corolla at T2, and some deterioration of stamens was visible at T6 (Figure 1). However, no significant differences were observed between T2 and T6 (Table 3). The pink striations on the white *P. odoratissimum* petals enhanced their hue during cold storage. Moreover, anthers were very delicate, detaching themselves from filaments after few hours of postharvest (personal observation). After 6 days of storage, the wrinkling of the petals and the detachment of a few petals were observed if handled with a consequent statistically significant decrease in the visual quality of the flowers (Table 3).

**TABLE 3:** Visual quality of flowers of *A. houstonianum*, *T. lemmonii*, *S. dorisiana*, and *P. odoratissimum* “Lemon,” at 0 (T0), 2 (T2), and 6 (T6) postharvest days (storage at 4°C).

		*A. houstonianum*	*T. lemmonii*	*S. dorisiana*	*P. odoratissimum* “Lemon”

Species	Days		*Visual quality*		
	0	4.00 ± 0.01^a^	4.00 ± 0.01^a^	4.00 ± 0.01^a^	4.00 ± 0.01^a^
	2	2.43 ± 0.31^b^	3.84 ± 0.09^a^	3.19 ± 0.33^b^	3.53 ± 0.19^a^
	6	1.50 ± 0.34^c^	3.75 ± 0.10^a^	2.89 ± 0.23^b^	2.67 ± 0.35^b^

*Data are shown as mean ± SE; *n* = 15. Different letters indicate statistically significant differences (*P* < 0.05; Tukey’s HSD test) between experimental points of each species.*

### Biochemical Profile

The first investigation performed on fresh flowers was the detection of membrane integrity in order to check their initial value and follow the change during postharvest. MDA content (TBARS assay) is a suitable marker to measure an increase in lipid peroxidation (Bailly et al., 1996) that is usually involved in the determination of cold tolerance (Zhuang et al., 1997). At T0, the content is different and peculiar for each EF examined (Table 3). Postharvest treatment at 4°C affected the membrane integrity in all examined flowers (Table 4). A rise in lipid peroxidation was observed in *A. houstonianum* and *P. odoratissimum* at T2, with an increase in MDA concentration (1.7- and 1.3-fold, respectively). At T6, cellular membrane damages were detected also in *T. lemmonii* and *S. dorisiana*, and the latter showed the highest increment in MDAe between T0 and T6 (4.4-fold).

**TABLE 4:** Lipid peroxidation (TBARS assay), total polyphenolics (TPC), flavonoids (TFC), anthocyanins, ascorbic acid content, and radical scavenging activity (DPPH) of *A. houstonianum*, *T. lemmonii*, *S. dorisiana*, and *P. odoratissimum* “Lemon” at 0 (T0), 2 (T2), and 6 (T6) postharvest days (storage at 4°C).

		TBARS assay (nmol MDAe/g FW)	Total anthocyanins (mg ME/g FW)	TFC (mg CE/g FW)	TPC (mg CE/g FW)	Total ascorbic acid (mg AsA_*TOT*_/g FW)	DPPH (IC_50_mg/ml)

Species	Days						
*A. houstonianum*	0	1.53 ± 0.10^b^	0.15 ± 0.01^a^	4.83 ± 0.16^a^	12.18 ± 0.27^a^	0.28 ± 0.03^b^	0.64 ± 0.01^b^
	2	2.60 ± 0.06^a^	0.14 ± 0.01^a^	4.28 ± 0.33^a^	11.69 ± 1.21^a^	0.49 ± 0.03^a^	0.86 ± 0.06^a^
	6	2.49 ± 0.12^a^	0.17 ± 0.03^a^	4.96 ± 0.13^a^	11.91 ± 0.14^a^	0.29 ± 0.03^b^	0.79 ± 0.01^a^
*T. lemmonii*	0	0.81 ± 0.04^b^	0.04 ± 0.00^b^	5.72 ± 0.17^b^	25.36 ± 0.33^a^	1.01 ± 0.01^b^	0.10 ± 0.01^b^
	2	0.67 ± 0.10^b^	0.11 ± 0.00^a^	6.53 ± 0.49^a^	24.54 ± 0.64^a^	1.25 ± 0.03^a^	0.17 ± 0.01^a^
	6	1.31 ± 0.07^a^	0.16 ± 0.05^a^	7.22 ± 0.14^a^	23.46 ± 1.22^a^	1.19 ± 0.01^a^	0.16 ± 0.01^a^
*S. dorisiana*	0	1.01 ± 0.08^b^	1.13 ± 0.07^b^	2.17 ± 0.16^b^	4.31 ± 0.29^b^	0.41 ± 0.00^b^	1.32 ± 0.03^a^
	2	1.12 ± 0.04^b^	1.40 ± 0.04^a^	3.23 ± 0.10^a^	4.96 ± 0.28^ab^	0.63 ± 0.01^a^	0.90 ± 0.07^b^
	6	4.43 ± 0.11^a^	1.55 ± 0.05^a^	3.33 ± 0.10^a^	5.58 ± 0.38^a^	0.43 ± 0.00^b^	1.06 ± 0.15^b^
*P. odoratissimum*	0	0.56 ± 0.06^c^	0.48 ± 0.03^b^	2.77 ± 0.01^c^	29.28 ± 0.14^a^	0.55 ± 0.02^b^	0.11 ± 0.01^a^
	2	0.73 ± 0.01^b^	0.50 ± 0.05^ab^	3.18 ± 0.02^b^	29.72 ± 0.18^a^	0.56 ± 0.02^b^	0.13 ± 0.01^a^
	6	1.26 ± 0.02^a^	0.65 ± 0.02^a^	5.51 ± 0.05^a^	26.78 ± 0.31^b^	0.80 ± 0.01^a^	0.06 ± 0.00^b^

*Data are shown as mean ± SE; *n* = 3. Different letters indicate statistically significant differences (*P* < 0.05; Tukey’s HSD test) between experimental points of each species.*

Blue, red, and purple pigmentations of flowers are mainly due to anthocyanins, and their content was examined in all samples. At the time of harvest (T0), the magenta *S. dorisiana* flowers showed the highest content of anthocyanins (1.13 mg/g FW), followed by those white-pink *P. odoratissimum* (0.48 mg/g FW). Limited amounts were observed in Lemmon’s marigold (0.04 mg/g FW) and *A. houstonianum* (0.15 mg/g FW) (Table 4). During the imposed postharvest condition, anthocyanins increased either at T2 or at T6 in the flowers with the exception of *A. houstonianum*, in which these metabolites were not altered up to the end of the experiment.

Total flavonoids content (TFC) was also investigated. At T0, the highest values were observed in *T. lemmonii* (5.72 mg/g FW), followed by *A. houstonianum* > *P. odoratissimum* > *S. dorisiana* (4.83, 2.77, and 2.17, respectively) (Table 4). During flower conservation, the flavonoid content strictly followed the trend already described for anthocyanins.

The class of polyphenols (including flavonoids) was measured for all experimented species and showed that at T0, high total polyphenols content (TPC) was observed in two flowers: *P. odoratissimum* (29.28 mg/g FW) and *T. lemmonii* (25.36 mg/g FW). *S. dorisiana* contained the lowest amount (4.31 mg/g FW) (Table 4). During cold storage, the examined flowers showed different trends. No change in the (TPC) was observed in *A. houstonianum* and *T. lemmonii*. In *P. odoratissimum*, a significant reduction was highlighted only at T6, at the end of storage condition (26.78 mg/g FW). On the other hand, *S. dorisiana* showed an opposite response to cold storage, displaying a progressive increase in TPC during the postharvest process (T0, 4.31 mg/g FW; T2, 4.96 mg/g FW, and T6, 5.58 mg/g FW).

Initial levels of total ascorbate (ASA_*TOT*_) are different between species. Lemmon’s marigold is the fresh flower with the highest content (1.01 mg/g FW), and *A. houstonianum* have the lowest one (0.28 mg/g FW) (Table 4). During conservation in plastic boxes at 4°C, the level of ASA_*TOT*_ increased in *T. lemmonii* at T2, without any reduction at T6. *P. odoratissimum* increased ASA_*TOT*_ value after 6 days of storage. ASA_*TOT*_ augmented significantly in *A. houstonianum* and *S. dorisiana* at T2 (1.5-fold) to decrease afterwards (T6), returning to the initial values.

The scavenger radical activity was determined with DPPH assay and expressed as IC_50_ (mg/ml). The highest antioxidant activity was detected in *T. lemmonii* and *P. odoratissimum* (0.10 and 0.11 mg/ml, respectively). Lower values were observed in the other two species. During cold storage, a strong increase in the antioxidant activity was detected in *S. dorisiana* between T0 (1.32 mg/ml) and T2 (0.90 mg/ml) and *P. odoratissimum* between T2 (0.13 mg/ml) and T6 (0.06 mg/ml). *A. houstonianum* and *T. lemmonii* showed a decrease in antioxidant activity at T2. Regardless of sampling, radical scavenger activity (DPPH assay) was negatively correlated with highest content of several antioxidants in flowers as reported in Table 5.

**TABLE 5:** Correlation between antioxidants compounds (polyphenols, flavonoids, anthocyanins, and ascorbic acid) in the flowers of four species (*A. houstonianum*, *T. lemmonii*, *S. dorisiana*, and *P. odoratissimum*) and the radical scavenger activity (DPPH assay).

Species	Antioxidant compounds	Equation	*R*^2^
*A. houstonianum*	Total polyphenols	*y* = −0.0964x + 1.8922	0.8675
*T. lemmonii*	Total polyphenols	*y* = −0.0215x + 0.6557	0.8103
*S. dorisiana*	Total anthocyanins	*y* = −0.9029x + 2.2889	0.7883
*S. dorisiana*	Total polyphenols	*y* = −0.2194x + 2.2498	0.6104
*P. odoratissimum*	Total flavonoids	*y* = −0.0205x + 0.1700	0.8325
*P. odoratissimum*	Total ascorbic acid	*y* = −0.2172x + 0.2313	0.8236
*P. odoratissimum*	Total anthocyanins	*y* = −0.2817x + 0.2485	0.7295

The nutritional value of edible flowers is also due to primary metabolites, such as sugars and proteins. Levels of soluble sugars (D-glucose, D-fructose, and sucrose) changed significantly depending on the examined species (Table 6). The highest sugar content in fresh flowers was determined in *P. odoratissimum*, while the lowest amount was observed in *A. houstonianum*. The greatest sucrose content was observed in *S. dorisiana* (4.4 mg/g FW), while *P. odoratissimum* underlined the major amount of hexoses content: glucose (10.41 mg/g FW) and fructose (6.09 mg/g FW). During postharvest condition, a strong decrease in sugars was observed in *P. odoratissimum*: glucose and sucrose concentrations dropped gradually from T0 to T6, while fructose decreased only at T6. On the other hand, levels of glucose and fructose in *S. dorisiana* increased over the period of storage, while sucrose decreased. Regarding *A. houstonianum*, no variation was observed in sucrose concentration all over the period of storage, while glucose and fructose content increased at T2, then decreased at the end of experiment. No alteration in the sucrose content was observed in *T. lemmonii* through the cold storage, while glucose and fructose decreased at T6. Considering the protein content, *A. houstonianum* contained 16.12% of crude protein (dry weight), followed by *T. lemmonii*, *P. odoratissimum*, and *S. dorisiana* (11.41, 6.43, and 5.75%, respectively). No statistically significant differences were observed among the four species analyzed throughout the cold storage (Table 6).

**TABLE 6:** Soluble sugars (D-glucose, sucrose, D-fructose) content and crude proteins of *A. houstonianum*, *T. lemmonii*, *S. dorisiana*, and *P. odoratissimum* “Lemon” at 0 (T0), 2 (T2), 6 (T6) postharvest days (storage at 4°C).

		D-GLUCOSE (MG/G FW)	D-fructose (mg/g FW)	Sucrose (mg/g FW)	Crude proteins (% DW)

Species	Days				
*A. houstonianum*	0	1.810.01^b^	1.130.03^b^	0.690.22^a^	16.191.12^a^
	2	2.420.06^a^	1.910.17^a^	0.620.26^a^	17.660.70^a^
	6	0.780.04^c^	0.560.04^c^	0.730.26^a^	17.610.38^a^
*T. lemmonii*	0	2.630.14^a^	2.530.12^a^	2.040.16^a^	11.410.11^a^
	2	2.280.01^b^	2.400.03^a^	2.100.15^a^	12.420.42^a^
	6	1.880.05^c^	2.110.04^b^	2.020.10^a^	12.300.34^a^
*S. dorisiana*	0	3.100.16^c^	4.060.26^c^	4.400.40^a^	5.750.29^a^
	2	4.990.03^b^	5.420.23^b^	2.550.07^b^	5.650.30^a^
	6	7.430.26^a^	7.360.19^a^	2.130.12^c^	4.400.18^a^
*P. odoratissimum*	0	10.410.64^a^	6.090.30^a^	3.170.03^a^	6.431.21^a^
	2	8.420.03^b^	5.440.31^a^	2.370.12^b^	7.590.16^a^
	6	5.870.25^c^	4.230.08^b^	1.560.09^c^	7.770.15^a^

*Data are shown as mean ± SE; *n* = 3. Different letters indicate statistically significant differences (*P* < 0.05; Tukey’s HSD test) between experimental points of each species.*

### Aroma Profile

More than 100 compounds were identified in the four studied flowers analyzed during different postharvest days even though the number of constituents differed from species and time of storage (Supplementary Table S1). The lowest number of compounds was noted in *T. lemmonii* (an average of 21 in the three postharvest periods), while this number was duplicated in *P. odoratissimum* (an average of 40). *A. houstonianum* showed β-caryophyllene (86) as the main compound in fresh flowers (31.6%), but its amount decreased by about 38% after 6 days of cold storage (19.5%). Germacrene D (98), the second main constituent at T0, pointed out the same trend and evidenced a lowering of 30% after 6 days (from 13.4 to 9.4% at T0 and T6, respectively). Both myrcene (10) and δ-2-carene (11) revealed a large slashing in their percentage (not < 87%) according to the storage time (reduction of 87.6 and 93.4% in myrcene and δ-2-carene, respectively). On the contrary, it is worthy to note the appearance of limonene (18) after 2 days of storage with high amount (30.5%), which was maintained at T6. Eleven new compounds appeared at this time with a percentage <1%, except for γ-terpinene (24) (3.4%). β-Caryophyllene (86), together with germacrene D (98), was also present in very good amount (23.0 and 10.9%, respectively). Limonene (18) was also the main compound after 6 days of storage followed by β-caryophyllene (26.1 and 19.6%, respectively). Noteworthy at the same time (T6) are the increase in precocene II value (118) (about 94%) and the disappearance of 14 compounds, 10 of which belong to sesquiterpene hydrocarbons.

The second species belonging to Asteraceae family, *T. lemmonii*, showed a completely different profile. The number of constituents increased from T0 to T2 (from 16 to 24, respectively) and maintained at T6 (24 constituents) (see Supplementary Table S1). Except at the time of conservation, almost all *T. lemmonii* samples were constituted by compounds belonging to monoterpenes (total monoterpenes ranging from 96.7 at T0 to 84.4% at T6, respectively). The fresh flowers were dominated by monoterpene hydrocarbons (63.3%), which were replaced by oxygenated monoterpenes in both later days, as explained by the decrease in both β-ocimene isomers. The *cis-* (21) and *trans-* (23) β-ocimene forms decreased of about 100% (from 17.9 and 43.2% at T0 to traces at T6, respectively), even though the latter one [(*E*)-β-ocimene] maintained the same percentage at both T0 and T2.

In this study, the Lamiaceae family was represented by *S. dorisiana* flowers. No difference was evinced in the number of constituents especially at the first two times with a slight decrease at T6. It is important to underline the enhancement of the total monoterpene percentage, which passed from 51.6 to more than 90% at both T2 and T6. Total sesquiterpenes decreased from 45.5% to around 8% at T2 and T6, respectively. Myrtenyl acetate (72), methyl perillate (82), and *trans*-α-bergamotene (89) evidenced a decline of their percentages of about 100% (from 9.1, 8.2, and 21.5% at T0 to traces at T6, respectively). On the other hand, *p*-cymene (17) increased (from 0 to 7% at T0 and T6, respectively) as well as α-pinene (5) (from 0 to more than 2.0% after 2 or 6 days of storage). 1,8-Cineole (20) evidenced a strange behavior at the first time of collection; 14.7% of the total composition disappeared at T2 and reappeared at T6 with a good amount (10.4%). This fluctuation was also noted in limonene content but with an increase in its percentage at T2 in comparison with T0 (from 12.6 to 41.2%, respectively) and then drop at T6 (24.8%).

*P. odoratissimum*, belonging to Geraniaceae family, was the unique species where the time of storage did not affect the number of the identified compounds (37, 40, and 42 at T0, T2, and T6, respectively). It is important to underline the scale-down of the total sesquiterpene amount (from 80.7% at T0 to 7.6% at T6, respectively) against a step-up of total monoterpenes (from 15.9% at T0 to 93.9% at T6, respectively). This depended on the decrease in the amounts of β-caryophyllene (86), germacrene D (98), and bicyclogermacrene (105) (from 23.2, 27.0, and 7.9%, respectively, at T0 to 2.2, 3.3, and 0.5%, respectively, T6). On the contrary, each of *p*-cymene, γ-terpinene (24), and α-pinene (5) undergone an increase in their amount from 0.3, 2.7%, and trace, respectively, at T0 to 15.4, 7.5, and 6.6% at T6, respectively.

**FIGURE 2:**
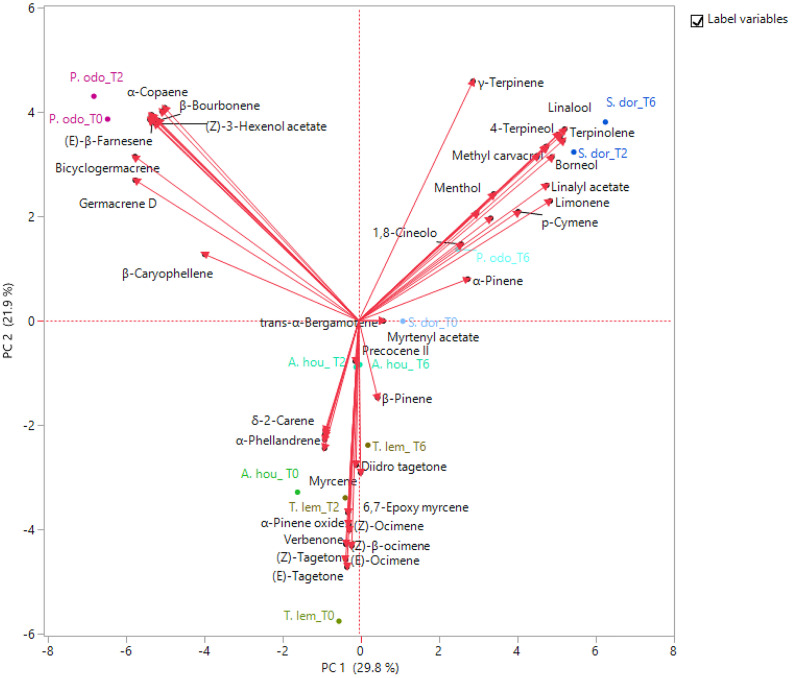
Principal component analysis (PCA) plot of volatile organic compounds (VOCs) emitted by the flowers of *A. houstonianum*, *T. lemmonii*, *S. dorisiana*, and *P. odoratissimum* “Lemon” collected at different times of postharvest (0, 2, and 6 days).

**FIGURE 3:**
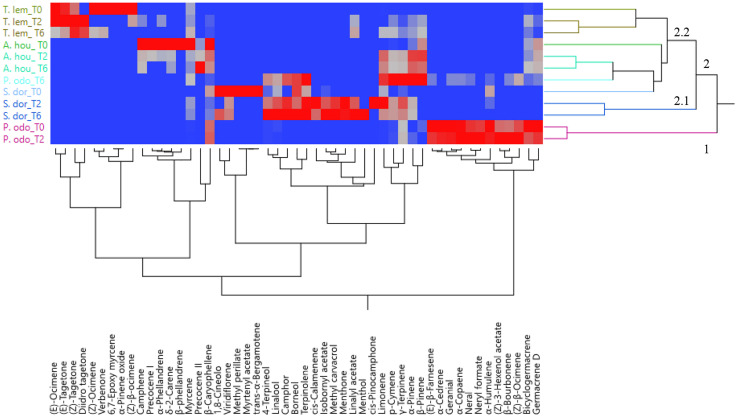
Heatmap of the two-way hierarchical cluster analysis (HCA) of volatile organic compounds (VOCs) emitted by the flowers of *A. houstorianum*, *T. lemmonii*, *S. dorisiana*, and *P. odoratissimum* “Lemon” collected at different times of postharvest (0, 2, and 6 days).

The PCA was used to examine the relative distribution of the studied samples, using the percentage of volatile compounds present in the amount ≥1%. PC1 and PC2 explained 51.7% of the variation (Figure 2). As shown in this figure, *P. odoratissimus* and *S. dorisiana* were clearly separated from the samples belonging to the Asteraceae family. *S. dorisiana* was located in the right quadrant (positive loading on PC1). Even though this position encompassed all the *Salvia* samples, a difference was noted between fresh flowers (T0) and those cold stored (T2 and T6). This was especially due to the presence of *trans*-α-bergamotene and myrtenyl acetate in fresh samples, which completely disappeared in T2 and T6. Both fresh and T2 flowers of *P. odoratissimum* were placed in the opposite quadrant (negative score along PC1). T6 instead showed a composition rich in monoterpene compounds, especially limonene and *p*-cymene, therefore *P. odoratissimum* at T6 is located in the same quadrant as *S. dorisiana*. *A. houstonianum* at T2 and T6 was placed exactly along the PC1 due to the amount of precocene II, which was the main compound that differed in these samples from the fresh flowers (T0) situated a bit far from the hub. The same trend was also shown in *T. lemmonii* where the fresh samples were far from the hub. For further characterization of the volatile samples, a two-way hierarchical cluster analysis (HCA) followed by heat map using the abundance of individual volatiles were performed. HCA dendrogram of *P. odoratissimum* differed at T0 and T2 (group 1) from all other the samples (group 2). The crucial compounds for their clustering were bicyclogermacrene and germacrene. Group 2 was further divided in two subgroups: 2.1 and 2.2. Subgroup 2.1 included only *S. dorisiana* at T2 and T6, while the subgroup 2.2 comprised all the remaining samples. *Salvia* sample at T0 was very close to the other *Salvia* samples analyzed at T2 and T6. This was explained by the fact that all the *Salvia* samples were rich in monoterpene compounds represented by methyl perillate and verbenone in fresh flowers that were substituted by other compounds like linalool, camphor, and 4-terpineol (see Figure 3).

The two-way PERMANOVA performed on the VOC emission showed that both factors (family membership and time of conservation) had no significant influence on the volatile composition (Table 7).

**TABLE 7:** Two-way PERMANOVA analysis (permutation N: 9,999).

Source	Sum of sqrs	df	Mean square	*F*	*P*
Family	0.84432	2	0.42215	0.55819	0.387
Time	0.59612	2	0.29805	0.3941	0.6251
Interaction	–0.62946	4	–0.15737	–0.20808	0.9911
Residual	2.26883	3	0.75628		
Total	3.0798	11			

## Discussion

### Visual Quality and Biochemical Profile During Cold Storage

The four selected species belong to different families (Asteraceae, Lamiaceae, and Geraniaceae). Flowers show different morphologies, as evidenced also between the two Asteraceae species (Figure 1), which could confer different adaptations to cold stress. Fresh flowers were also characterized by different nutraceutical properties, due to the content of sugars, proteins, and antioxidant compounds, and this work reported for the first time their biochemical profile. At the time of harvest (T0), the highest sugar content was observed in *P. odoratissimum*, followed by *S. dorisiana*. The Lamiaceae family seems to produce flowers with high sugar content, probably due to nectar production (Maèukanović-Jocić et al., 2011), as recently confirmed in other *Salvia* species (Marchioni et al., 2020). *P. odoratissimum* showed also the highest total polyphenols content (TPC); similar values were obtained in *T. lemmonii*, which contained also the highest amount of ascorbic acid (ASA_*TOT*_). High vitamin C content seems to be peculiar for this genus, as also confirmed in *T. tenuifolia* (241.20 mg/100 g FW) (Grzeszczuk et al., 2016). *A. houstonianum* is the unique species previously analyzed for its anthocyanin content and antioxidant activity (Benvenuti et al., 2016). In this work, other compounds were measured, highlighting the utmost crude protein content, compared to the other species under evaluation.

Postharvest studies are carried out on ornamental potted plants or cut flowers with great economical values (Cavaiuolo et al., 2013; Rogers, 2013; Ferrante et al., 2015). The conservation and flower quality are essential requirements. Therefore, the strategy is often focused on the addition of molecules able to delay the senescence process (Cocetta et al., 2017; Silva et al., 2018; Rabiza-Świder et al., 2020), and different techniques are available for the prolongation of visual appearance and flowers’ shelf-life (Rani and Singh, 2014). In comparison with ornamental flowers, EFs are sold without stem in small packages made of various materials. Their postharvest physiology received less attention, and only few papers are published on this topic until now (Kelley et al., 2003; Landi et al., 2015, 2017; Pal et al., 2016; Demasi et al., 2020).

Currently, cold storage is a common nontoxic treatment used to prolong the shelf-life (Landi et al., 2017; Fernandes et al., 2019b). This treatment first affects the flowers’ visual quality, and hydration appears as an important characteristic to maintain the shape of flowers once bloomed (Ahmad and Tahir, 2016). In the present work, the conservation of cut stalk-less flowers in plastic boxes at 4°C for 6 days resulted in an early visible dehydration. The reduction in cell turgidity was confirmed in *P. odoratissimum*, since their petals looked crumpled already at T2, and the negative effect of water loss was evident at T6 (Tables 2, 3).

Within the Asteraceae family, discrepancies were observed; the morphology of *A. houstonianum* flowers strongly compromised the storage due to dehydration and color change. By contrast, *T. lemmonii* showed limited signs of dehydration, similarly, as observed in *T. patula* after 7 postharvest days (Chrysargyris et al., 2018). The broad *Salvia* genus highlighted differences between single species; *S. dorisiana* showed limited weight loss and discoloration, while in other *Salvia* species, divergent behavior was observed (Landi et al., 2015, 2017).

The aesthetic quality of flowers (score ratings) during cold storage may reflect some parameters as weight loss and cellular membrane damage, with a consequent impairment of cell biochemical and biophysical properties, due to changes in lipid composition and polyunsaturated fatty acids peroxidation (Thompson et al., 1998; Chakrabarty et al., 2009). In the present work, the change in rating scores was mainly due to MDA concentration in *S. dorisiana*, while in *P. odoratissimum* and *A. houstonianum*, it was probably linked to both parameters and flower morphology.

The presence of specific secondary metabolites contributes to flower pigmentation and thus their visual quality. Anthocyanins, along with other flavonoids and phenolic compounds, are considered the most abundant and representative bioactive and antioxidant metabolites that characterize fresh flowers, including the edible ones (Mlcek and Rop, 2011; Lu et al., 2016). The polyphenols content (TPC) is also related to the browning process and represent the molecules that neutralize reactive oxygen species (ROS), produced by both cold treatment and the detachment of flowers from their mother plant (Rogers, 2012; Cavaiuolo et al., 2013; Darras, 2020).

The scavenger activity detected by DPPH assay (IC_50_) confirmed the role played by these compounds during the cold storage, which allow to maintain the nutritional quality of flowers and therefore delay their senescence.

*Pelargonium odoratissimum* “Lemon” and *S. dorisiana* showed similar changes in these substances during the cold storage. This was evident by the increased amount of flavonoids, anthocyanins, and radical scavenging activity at T6. However, the polyphenols content diverged due to the different coloration of flowers, changing, therefore, the role played by these metabolites during storage. Nevertheless, the observed decrease in IC_50_ values during cold storage in both species was positively affected by an increase in total anthocyanin content, as demonstrated by the correlation analysis (Table 6). In the literature, the positive influence of low temperature on flower anthocyanin content has been already observed, e.g., *Viola cornuta* (Demasi et al., 2020), *Gerbera hybrida* (Naing et al., 2018), *Salvia* hybrid (Landi et al., 2015), and *Petunia hybrida* (Shvarts et al., 1997). Despite this clear evidence, the regulatory mechanisms in flowers are under debate. Until now, the induction of the expression of anthocyanins biosynthetic genes and two transcriptional factors (MYB10 and MYC1) has been demonstrated in *G. hybrida* flowers (Naing et al., 2018). A similar transcriptional regulation was observed also in some fruits, such as grapes (Sanchez-Ballesta et al., 2007), oranges (Crifò et al., 2012; Lo Piero et al., 2005), and red fleshed kiwifruit (Li et al., 2017).

Ascorbic acid is another antioxidant molecule able to preserve the quality of flowers. The examined flowers increased the ASA_*TOT*_ content during the cold storage. Nevertheless, a negative correlation between ascorbic acid and antioxidant activity was observed only in *P. odoratissimum* “Lemon.” However, the mechanism of ASA involvement during prolonged refrigeration is very complex. This demonstrated the influence of the Halliwell/Foyer/Asada (HFA) cycle, which is responsible for tissue survival under chilling temperatures (Wise, 1995). Further studies on gene regulation of MDHAR and DHAR proteins will provide better knowledge about the interaction between the activity of these enzyme with ASA level, as already demonstrated (Ferrante et al., 2015).

The recent study on cold storage of *Begonia semperflorens* and *Viola cornuta* flowers of mixed colors highlighted the different aptitude to postharvest between the species (Demasi et al., 2020).

This was confirmed by the two Asteraceae species, *T. lemmonii* and *A. houstonianum*. After the dehydration process, the concentration of bioactive molecules in blue flowers did not change (referred to fresh weight), so it was assumed that *A. houstonianum* suffered the cold stress mainly due to the weight loss and its flower morphology. However, the prolonged postharvest did not influence the amount of bioactive compounds. On the other hand, *T. lemmonii* maintained a good visual quality up to T6 and tolerated the cold storage by the rise in flavonoids, anthocyanins, and ASA_*TOT*_ amounts.

Within the examination of the metabolic rearrangement during cold storage of detached flowers, it is important to underline that low temperature delays respiration and transpiration rates, associated with the senescence process (Nichols, 1973; Fernandes et al., 2019b; Darras, 2020). Sugars are substrate of the cellular respiration; meanwhile, soluble sugars are an important flower nutritional component that represents a good characteristic for the choice of edible flowers. A balanced mixture of these metabolites (fructose, glucose, and sucrose) is present in petals and nectar (Mlcek and Rop, 2011). The fragrance is often related to the transport of sucrose from the mother plant to the flowers, where it contributes to the synthesis of essential oils, as seen in *Rosa damascena* (Pogorelskaya et al., 1980). In this work, the variation in fructose, glucose, and sucrose content was examined. *T. lemmonii* and *A. houstonianum* were not or slowly affected by cold stress, while *Pelargonium* decreased generally the content. *S. dorisiana* increased the availability of hexoses during stress, which could be related to a probably worst adaptation of *Salvia* to cold treatment. Indeed, the rate of sugar catabolism represents one of the factors determining the flower longevity (Nichols, 1973). This behavior could be also dependent on the ethylene action during senescence because ethylene is known to affect the amount of soluble sugars in ethylene-sensitive flowers (Paliyath et al., 2008). Further investigation will define the role of ethylene in these flowers.

This biodiversity of flower morphology and attitude to cold storage suggest that the choice of flower species is important to preserve the flower visual aspect and water loss, therefore delaying the onset of senescence process. The rearrangement of metabolic compounds during conservation followed the known changes for cold stress and subsequently the detachment of mother plants. *T. lemmonii* seems to be the flower with the best attitude to maintain bioactive molecules during cold storage, since the metabolic changes normally occurring in the postharvest process are limited.

### Aroma Profile

Owing to the lack of investigation on the VOCs of *A. houstonianum* and *T. lemmonii*, only few reports dedicated to the essential oil (EO) composition rather than on the spontaneous emission were analyzed. *A. houstonianum* was studied by Kurade et al. (2010), who confirmed the presence of chromenes such as precocene-I (22.45%) and precocene-II (52.64%) as main compounds in the EO of the aerial parts from plants collected in Palampur region of India. These two compounds were also present in the current study but in different amount. Another paper performed on a fraction of the EO obtained from the aerial parts of *A. houstonianum* underlined precocene-II (48.82%) as the main compound together with β-caryophyllene (20.80%) (Ramadan et al., 2007). This latter constituent was also found in the aroma profile of the titled species where it represented at least 20% of the total identified fraction. The Ivory Coast flowers EO of *Ageratum conyzoides*, a plant belonging to the same genus of the species studied herein, was characterized by precocene-I (58.8%) and β-caryophyllene (15.2%) (Kouame et al., 2018), while the EO obtained from the aerial parts of the same species from Nepal revealed both prococene-I (61.7%) and prococene-II (23.5%) as the most important constituent (Satyal et al., 2018).

The investigation on the spontaneous emission of *T. lemmonii* is not exhaustive, too, although a study on both volatile and essential oil composition of other species of the same genus such as *T. minuta* and *T. patula* had been slightly extensive. *T. lemmonii* EO from Camden, Delaware (United States) was investigated by in Tucker and Maciarello, 1996. They pointed out dihydrotagetone (42.52%), (*E*)-tagetone (16.10%), and (*E*)-ocimenone [also called (*E*)-tagetenone, 14.18%] as dominant constituents. All these compounds were present in the VOC composition even though with different amount. Dihydrotagetone showed a lower percentage after 2 days of storage (30.79% at T2) and decreased to half amount at T6 (24.63%); as with (*E*)-tagetenone (7.75%), in comparison with those reported by Tucker and coworkers, (*E*)-tagetone was present in very low amount. Another species of *Tagetes* from Argentina, *T. minuta*, was analyzed by HS-SPME and the inflorescences evidenced both *trans*- (26.1%) and *cis*-tagetenone (17.8%) as major components, while the cultivated one (Ghiasvand et al., 2011) showed the following as more important: *trans*-caryophyllene (19.7%), piperitenone oxide (12.3%), (*E*)-tagetenone (10.3%), and germacrene D (10.0%). These results differed from the volatile composition found in this work. Prakash et al. (2012) focused their study on the volatile emission of *T. patula* collected in Lucknow (India), where they found a completely different profile with the domination of (*Z*)-β-ocimene (21.8%) after 60 min for contact PDMS/DVS/CAR fiber of plucked capitula, terpinolene (16.7%), and δ-elemene (22.5%).

Only one work on the volatile composition of *S. dorisiana* was reported by Ascrizzi et al. (2017), where the authors underlined a profile rich in limonene (65.1%) followed by comparable percentage of both methyl pyrollate and myrcene (6.9 and 6.3%, respectively). These three constituents were also found in this study even though with different amounts. The newest work on EO composition of this species date back to 2012 where the team of Professor Conti et al. (2012) found a good amount of aromadendrene (25.7%), α-gurjenene (17.0%), and limonene (7.6%). Except for limonene, which was present in high amount also in the aroma profile investigated herein, the other two mentioned compounds were almost absent.

*Pelargonium odoratissimum* is a plant for which little technical and scientific knowledge exists. Few studies reported on the biogenic volatile organic compounds, especially in other species belonging to the same genus, as in the paper of Deng et al. (2004) who evaluated the aroma profile of *P. hortorum* leaves grown in greenhouse at the Shanghai Institute of Plant Physiology and Ecology. Myrcene (39.8%) and caryophyllene (32.1%) were found as major constituents, completely different from the herein composition. A new recent study investigated the EO composition of *P. odoratissimum*, where citronellol (33.0%), geraniol (15.4%), and citronellyl formate (6.8%) were identified as principal compounds (Orchard et al., 2019), while citronellol (30.1%), isomenthone (16.2%), and citronellyl formate (9.1%) characterized the Italian *P. odoratissimum* EO (Benelli et al., 2018). Another paper dates back to 2011 (Andrade et al., 2011), which evidenced that the leaf EO from Brazil samples was characterized by a methyleugenol chemotype since this was almost the unique constituent (96.8% of the identified fraction).

In this study, a difference in VOC emission was obtained during the time of storage of each flower species. The literature reported the chilling effect in VOC emission especially in the fruits (tomato: Farneti et al., 2015; peach: Brizzolara and Tonutti, 2017; Brizzolara et al., 2018). Unfortunately, these works were limited to report changes in aromatic profile, without including the effect of chilling in the cellular mechanism related to the VOC emission. Furthermore, the VOC composition was completely different from the aromatic compounds detected in the four studied EFs. Nevertheless, some volatiles, present in *A. houstonianum* and *P. odoratissimum*, such as farnesene, caryophyllene, and germacrene, were present in high amount in the headspace of fresh flowers but largely decreased after chilling storage. This might result from a short atmospheric chemical lifetime of terpenoid compounds as reported in *Jasminum sambac* (Zhou et al., 2019). Furthermore, the high amount of limonene (>25%) found in *A. houstonianum* may be justified by a decrease in myrcene amount due to cellular rearrangement (Kozioł et al., 2014). Moreover, limonene, which is considered as a specific cold-induced response in star ruby red grapefruit (Lado et al., 2019), probably plays the same role in both *S. dorisiana* and *T. lemmonii*, where an increase in its percentage was observed, even though in the latter species, this increase did not persist after 6 days of storage. In all the studied species, a considerable increase in monoterpene was noted, which allows us to hypothesize that emission of terpenes was most likely a response to chilling stress. A similar behavior was noted by Tietel et al. (2012) during the investigation on the effect of temperature on mandarin fruits.

## Conclusion

Edible flowers are considered a new source of functional food, and in the last years, many researchers focused their attention on this topic in relation to the increasing consumers and market demand. Within the four examined species, *T. lemmonii* appears as the most interesting one because of its high ASA content. Moreover, it highlighted the longest shelf-life, as demonstrated by the conservation of phytonutritional and aromatic profiles. On the other hand, *A. houstonianum* and *P. odoratissimum* should be consumed as soon as possible after their harvest or sold as potted plants. For these two species, further postharvest strategies should be tested to maintain their nutraceutical characteristics.

## Data Availability Statement

The raw data supporting the conclusion of this article will be made available by the authors, without undue reservation.

## Author Contributions

LaP: conceptualization. BN and IM: data curation. BN, IM, and BF: formal analysis. BR and LaP: funding acquisition. BN, IM, BR, AC, and LuP: investigation. LuP, LaP, and BN: methodology. BR: project administration. BR, LuP, and LaP: supervision. BN, IM, LuP, and LaP: writing—original draft. BN, IM, BR, AC, LuP, and LaP: writing—review and editing. All authors contributed to the article and approved the submitted version.

## Conflict of Interest

The authors declare that the research was conducted in the absence of any commercial or financial relationships that could be construed as a potential conflict of interest.
