# Supplementary material for: Phytonutritional Content and Aroma Profile Changes During Postharvest Storage of Edible Flowers
Source: Front Plant Sci. 2020 Nov 27;11:590968. doi: 10.3389/fpls.2020.590968 (PMC7731506; doi:10.3389/fpls.2020.590968)
Supplement: Supplementary file 1 [file Table_1.DOCX]

Supplementary Material

# Supplementary Tables

Table S1. Aroma profile of *A. houstonianum*, *T. lemmonii*, *S. dorisiana*, and *P. odoratissimum* detected by headspace solid phase microextraction, HS-SPME) in three different times of post-harvest (day 0, 2- and 6-). Data are reported as mean ±SD (n=3).

|  |  |  |  | **Asteracea** | | | **Laminaceae** | | | | | | **Geraniaceae** | | |
| --- | --- | --- | --- | --- | --- | --- | --- | --- | --- | --- | --- | --- | --- | --- | --- |
|  |  |  |  | ***A. houstonianum*** | | | ***T. lemmonii*** | | | ***S. dorisiana*** | | | ***P. odoratissimum*** | | |
|  |  |  |  | **Relative content (%)** | | | | | | | | | | | |
|  | **Compounds** | **Class** | **LRI^§^** | **0** | **2** | **6** | **0** | **2** | **6** | **0** | **2** | **6** | **0** | **2** | **6** |
| **1** | (*E*)-3-Hexenen-1-ol | nt | 851 | - | - | - | - | - | - | - | - | - | 0.25±0.08 | 0.91±0.61 | - |
| **2** | 1-Hexanol | nt | 867 | - | - | - | - | - | - | - | - | - | 0.07±0.00 | 0.49±0.32 | - |
| **3** | Triciclene | mh | 926 | - | - | - | - | - | - | - | - | - | 0.08±0.00 | 0.31±0.01 | - |
| **4** | α-Thujene | mh | 932 | - | 0.30±0.02 | 0.25±0.02 | - | - | - | - | - | - | 0.19±0.01 | 0.62±0.05 | 0.40±0.17 |
| **5** | α-Pinene | mh | 939 | 0.77±0.35 | 5.54±0.14 | 4.85±0.02 | tr | 1.05±0.07 | 1.39±0.26 | - | 2.60±0.14 | 2.34±0.05 | tr | 0.49±0.02 | 6.55±0.20 |
| **6** | Camphene | mh | 953 | 5.08±2.22 | 1.09±0.16 | 0.82±0.06 | - | 0.29±0.06 | tr | 0.07±0.00 | 0.17±0.00 | tr | tr | tr | 0.77±0.03 |
| **7** | Sabinene | mh | 976 | - | 0.54±0.03 | 0.42±0.02 | tr | 0.28±0.15 | 0.20±0.01 | - | 0.19±0.00 | tr | 0.44±0.14 | 0.55±0.07 | - |
| **8** | β-Pinene | mh | 980 | 1.12±0.51 | 1.72±0.11 | 1.36±0.04 | tr | 0.44±0.07 | 0.60±0.17 | 0.12±0.00 | 1.39±0.34 | 0.55±0.03 | 0.15±0.01 | tr | 2.00±0.42 |
| **9** | 6-methyl-5-heptan-2-one | nt | 985 | - | - | - | - | - | - | 0.13±0.00 | - | - | tr | 0.05±0.00 | - |
| **10** | Myrcene | mh | 991 | 12.89±6.16 | 2.13±0.49 | 1.60±0.27 | 2.06±0.05 | 1.74±0.05 | 3.75±0.55 | 0.35±0.00 | 1.84±0.21 | 1.55±0.01 | 0.41±0.05 | 0.38±0.07 | 2.75±0.10 |
| **11** | δ-2-Carene | mh | 1001 | 11.74±3.77 | 1.00±0.06 | 0.77±0.31 | - | - | - | - | - | - | - | - | - |
| **12** | (*Z*)-3-Hexenol acetate | nt | 1004 | - | - | - | - | - | - | - | - | - | 1.31±0.37 | 2.15±0.14 | - |
| **13** | α-Phellandrene | mh | 1005 | 5.05±2.52 | 0.40±0.11 | - | - | 0.28±0.08 | - | - | - | - | - | - | 0.30±0.06 |
| ***14*** | *n*-Hexyl acetate | nt | 1008 | - | - | - | - | - | - | - | - | - | tr | tr | - |
| **15** | δ-3-Carene | mh | 1011 | - | 0.45±0.08 | - | - | tr | - | - | - | - | tr | tr | 0.72±0.02 |
| **16** | α-Terpinene | mh | 1018 | - | 0.89±0.08 | 0.75±0.02 | - | 0.60±0.32 | 0.50±0.10 | 0.34±0.01 | 1.08±0.02 | 0.93±0.02 | 0.26±0.05 | 0.36±0.05 | - |
| ***17*** | *p*-Cymene | mh | 1026 | 0.16±0.4 | 4.53±0.52 | 3.17±0.09 | - | 1.72±0.04 | 3.37±0.83 | - | 6.97±0.67 | 6.99±0.05 | 0.27±0.08 | 0.38±0.03 | 15.36±3.75 |
| **18** | Limonene | mh | 1030 | - | 30.45±1.85 | 26.08±0.15 | 0.17±0.02 | - | 14.40±0.78 | 12.63±0.10 | 41.18±2.42 | 24.78±0.33 | 0.31±0.08 | 0.33±0.04 | 36.79±2.24 |
| **19** | β-Phellandrene | mh | 1031 | 1.95±0.79 | - | - | - | - | - | - | - | - | - | - | - |
| **20** | 1,8-Cineole | om | 1033 | - | - | - | - | - | - | 14.66±0.06 | tr | 10.44±0.00 | - | - | - |
| **21** | (*Z*)-β-Ocimene | mh | 1037 | - | 0.09±0.00 | 0.14±0.01 | 17.90±0.09 | 3.92±0.56 | tr | - | 0.19±0.07 | tr | 1.98±0.59 | 2.58±0.16 | 0.81±0.15 |
| **22** | Di-hydro tagetone | om | 1047 | - | - | - | tr | 30.79±0.31 | 24.63±0.93 | - | - | - | - | - | - |
| **23** | (*E*)-β-Ocimene | mh | 1050 | - | 0,05±0.00 | tr | 43.21±0.05 | 43.31±0.05 | tr | - | 0.59±0.01 | tr | 0.26±0.11 | 0.32±0.01 | 0.37±0.01 |
| **24** | γ-Terpinene | mh | 1062 | - | 3.38±0.26 | 2.96±0.04 | - | 0.34±.00 | 1.27±0.06 | 0.55±0.05 | 6.02±0.09 | 4.66±0.23 | 2.65±0.77 | 2.95±0.17 | 7.54±0.86 |
| **25** | Borneol | om | 1065 | 0.39±0.02 | 0.21±0.02 | tr | - | - | - | 1.48±0.16 | 1.20±0.01 | 1.75±0.09 | - | - | 1.46±0.32 |
| **26** | *cis*-Sabinene hydrate | om | 1068 | - | - | - | - | - | - | 0.37±0.04 | - | - | - | - | - |
| ***28*** | *trans*-Linalool oxide | om | 1088 | - | - | - | - | - | - | - | - | - | - | - | 0.18±0.00 |
| **27** | Terpinolene | mh | 1089 | 0.16±0.01 | 0.64±0.16 | 0.69±0.05 | - | 0.36±0.16 | 0.52±0.03 | 0.81±0.09 | 1.83±0.16 | 1.84±0.17 | tr | tr | 1.84±0.10 |
| **29** | 6,7-Epoxy myrcene | om | 1090 | - | - | - | 1.58±0.17 | - | - | - | - | - | - | - | - |
| ***30*** | α-Pinene oxide | om | 1095 | - | - | - | 3.33±0.10 | tr | - | - | - | - | - | - | - |
| **31** | Linalool | om | 1098 | - | 0.07±0.00 | - | 0.87±0.14 | 1.14±0.03 | 0.50±0.01 | 1.56±0.20 | 7.61±1.69 | 10.21±0.45 | 0.16±0.01 | 0.05±0.00 | 3.71±1.29 |
| **32** | *iso*-pentyl 2-methyl butyrate | nt | 1099 | - | - | - | - | - | - | 1.46±0.27 | - | - | - | - | - |
| **33** | *n*-Nonanal | nt | 1101 | tr | 0.15±0.00 | 0.59±0.05 | - | tr | - | - | - | - | 0.14±0.01 | tr | - |
| ***34*** | (*E*)-4,8-dimethylnona-1,3,7-triene | mh | 1113 | 0.05±0.00 | - | - | - | - | - | - | - | - | - | - | - |
| ***35*** | *trans*-*p*-mentha-2,8-dien-1-ol | om | 1120 | - | - | - | 0.38±0.08 | - | - | - | - | - | - | - | - |
| **36** | Chrysanthenone | om | 1123 | - | - | - | 0.55±0.06 | - | - | - | - | - | - | - | - |
| ***37*** | *allo*-cimene | om | 1129 | - | - | - | 1.43±0.18 | - | tr | - | - | - | - | - | - |
| **38** | (*Z*)-Myroxide | om | 1132 | - | - | - | 0.98±0.07 | - | - | - | - | - | - | - | - |
| ***39*** | *trans*-Pinocarveol | om | 1139 | - | - | - | - | - | - | - | 0.60±0.11 | 0.73±0.04 | - | - | 0.34±0.04 |
| **40** | Camphor | om | 1143 | tr | 0.42±0.16 | 0.86±0.13 | - | 1.06±0.06 | 0.51±0.01 | - | 3.35±0.31 | 3.75±0.07 | - | - | 2.79±0.71 |
| ***41*** | (*E*)-Tagetone | om | 1145 | - | - | - | 2.82±0.26 | 3.06±0.20 | 1.22±0.13 | - | - | - | - | - | - |
| **42** | (*Z*)-Tagetone | om | 1149 | - | - | - | 19.92±0.09 | 31.49±0.28 | 29.24±0.34 | - | - | - | - | - | - |
| **43** | Menthone | om | 1154 | - | - | - | - | - | - | - | 1.62±0.50 | 1.56±0.40 | - | - | 1.04±0.05 |
| **44** | *trans*-Pinocamphone | om | 1160 | - | - | - | - | - | - | - | - | - | - | - | 0.30±0.00 |
| **45** | Pinocarvone | om | 1165 | - | - | - | - | - | - | - | 0.21±0.00 | - | - | - | - |
| ***46*** | Menthol | om | 1171 | - | - | - | - | - | - | - | - | 2.46±0.14 | - | - | 0.59±0.03 |
| **47** | Pinocamphone | om | 1173 | - | - | - | - | - | - | 0.43±0.03 | 1.61±0.19 | - | - | - | - |
| **48** | 4-Terpineol | om | 1177 | - | - | - | - | tr | 0.27±0.00 | 0.28±0.05 | 3.90±0.85 | 6.84±0.08 | 0.06±0.00 | 0.05±0.00 | 3.29±0.04 |
| **49** | α-Terpineol | om | 1189 | - | - | - | - | - | - | 0.30±0.05 | 0.21±0.09 | tr | 0.08±0.00 | tr | - |
| **50** | Methyl chavicol | pp | 1195 | - | - | - | - | - | - | - | - | - | - | - | 0.61±0.01 |
| **51** | *n*-Decanale | nt | 1204 | 0.08±0.00 | tr | 0.67±0.03 | - | - | - | 0.49±0.31 | - | - | 0.14±0.00 | 0.16±0.02 | - |
| **52** | Verbenone | om | 1204 | - | - | - | 1.53±0.06 | - | 0.37±0.03 | - | - | - | - | - | tr |
| **53** | Octanol acetate | nt | 1211 | - | - | - | - | - | - | 0.15±0.04 | - | - | - | - | - |
| ***54*** | Citronellol | om | 1226 | - | - | - | - | - | - | - | - | - | - | - | 0.12±0.01 |
| **55** | (*Z*)-Tagetenone | om | 1229 | - | - | - | - | tr | 0.89±0.11 | - | - | - | - | - | - |
| **56** | Nerol | om | 1230 | - | - | - | - | - | - | - | - | - | 0.68±0.26 | 0.61±0.09 | - |
| **57** | *iso*-bornyl formate | om | 1233 | 0.48±0.05 | - | - | - | - | - | - | - | - | - | - | - |
| **58** | Butanoic acid, 3-methyl-3-hexen-1-yl ester | nt | 1238 | - | - | - | - | - | - | 0.07±0.00 | - | - | - | - | - |
| **59** | (*E*)-Tagetenone | om | 1239 | - | - | - | - | 0.24±0.04 | 7.75±0.50 | - | - | - | - | - | - |
| ***60*** | Neral | om | 1240 | - | - | - | - | - | - | - | - | - | 2.47±0.34 | 2.89±0.16 | 0.17±0.02 |
| **61** | Hexyl-3-methyl butanoate | nt | 1243 | - | - | - | - | - | - | 0.15±0.03 | - | - | - | - | - |
| **62** | Methyl carvacrol | om | 1244 | - | 0.20±0.05 | - | - | - | - | - | 2.06±0.23 | 2.41±0.09 | - | - | 0.14±0.02 |
| **63** | Carvacrol methyl ether | om | 1245 | - | - | - | - | - | - | - | - | - | - | - | 0.75±0.05 |
| **64** | Geraniol | om | 1256 | - | - | - | - | - | - | - | - | - | 0.59±0.44 | 0.54±0.07 | - |
| **65** | Linalyl acetate | om | 1257 | - | 0.31±0.03 | 0.41±0.01 | - | 0.78±0.07 | 1.90±0.02 | - | 3.75±0.06 | 5.24±0.26 | - | - | 1.47±0.01 |
| **66** | Geranial | om | 1270 | - | - | - | - | - | - | - | - | - | 3.11±0.15 | 2.92±0.05 | - |
| **67** | (*E*)- Anethone | pp | 1283 | - | - | - | - | - | - | - | - | - | - | - | 0.12±0.01 |
| **68** | *iso*-bornyl acetate | om | 1285 | 1.20±0.14 | 0.21±0.01 | - | - | tr | 0.21±0.00 | 0.06±0.00 | 1.88±0.36 | 2.68±0.04 | - | - | 0.92±0.02 |
| **69** | Methyl acetate | om | 1294 | - | - | - | - | - | - | - | - | - | - | - | 0.14±0.02 |
| **70** | Neryl formate | om | 1307 | - | - | - | - | - | - | - | - | - | 1.41±0.39 | 1.72±0.23 | - |
| **71** | Geranyl formate | om | 1322 | - | - | - | - | - | - | - | - | - | - | - | 0.14±0.02 |
| ***72*** | Myrtenyl acetate | om | 1325 | - | - | - | - | - | - | 9.08±0.82 | - | - | - | - | - |
| **73** | 2,3,5,8-Tetramethyl decane | nt | 1328 | - | 0.20±0.05 | - | - | - | - | - | - | - | - | - | - |
| **74** | δ-Elemene | sh | 1339 | 0.26±0.16 | - | - | tr | - | - | - | - | - | 0.53±0.06 | 0.26±0.15 | - |
| **75** | α-Longipinene | sh | 1351 | 0.51±0.02 | 0,20±0.04 | 0.39±0.02 | - | - | - | - | - | - | 0.23±0.04 | 0.27±0.11 | 0.11±0.01 |
| **76** | α-Cubebene | sh | 1353 | - | - | - | - | - | - | - | - | - | - | - | 0.12±0.01 |
| **77** | Neryl acetate | om | 1365 | 0.08±0.00 | - | - | - | - | - | - | - | - | - | - | - |
| **78** | α-Copaene | sh | 1376 | - | 0.08±0.00 | - | - | tr | tr | 0.17±0.06 | 0.61±0.05 | 0.29±0.01 | 1.86±0.10 | 1.87±0.17 | 0.16±0.06 |
| **79** | β-Bourbonene | sh | 1384 | 0.41±0.09 | 0.14±0.04 | - | - | - | - | - | - | - | 1.06±0.16 | 1.82±0.06 | 0.12±0.01 |
| **80** | β-Cubebene | sh | 1388 | 0.13±0.02 | 0.25±0.03 | - | - | - | - | - | - | - | 0.44±0.23 | 0.23±0.03 | tr |
| **81** | β-Elemene | sh | 1391 | 0.39±0.26 | 0.16±0.02 | - | - | - | - | - | - | - | 0.24±0.05 | 0.21±0.02 | tr |
| **82** | Methyl perillate | om | 1394 | - | - | - | - | - | - | 8.25±1.92 | - | - | - | - | - |
| **83** | α-Gurjunene | sh | 1409 | - | - | - | - | - | - | 0.67±0.07 | - | tr | - | - | - |
| **84** | α-Cedrene | sh | 1412 | - | - | - | - | - | - | - | - | - | 1.49±0.08 | 1.37±0.02 | - |
| **85** | *cis*-α-Bergamotene | sh | 1415 | - | - | - | - | - | - | 0.63±0.01 | - | - | - | - | - |
| **86** | β-Caryophellene | sh | 1419 | 31.57±4.15 | 23.02±1.31 | 19.55±0.21 | - | 2.60±0.19 | 1.49±0.38 | 17.95±0.28 | 3.89±0.01 | 5.59±0.28 | 23.23±1.73 | 24.33±0.30 | 2.22±0.11 |
| **87** | β-Copaene | sh | 1428 | 0.27±0.12 | 0.20±0.04 | - | - | - | - | - | - | - | 0.77±0.20 | 1.21±0.21 | tr |
| **88** | α-Guaiene | sh | 1437 | - | - | - | - | - | - | - | 0.13±0.00 | - | 0.38±0.06 | 0.29±0.13 | tr |
| ***89*** | *trans*-α-Bergamotene | sh | 1439 | 0.05±0.00 | - | - | - | - | - | 21.45±0.72 | - | - | 0.22±0.01 | 0.48±0.30 | - |
| **90** | α-Aromandrene | sh | 1440 | 0.12±0.01 | 0.23±0.04 | 0.12±0.01 | - | - | - | - | - | - | - | - | - |
| **91** | Aromadendrene | sh | 1442 | - | - | - | - | - | - | - | tr | - | - | - | 0.13±0.02 |
| **92** | α-Humulene | sh | 1454 | 1.05±0.42 | 0.60±0.01 | 0.58±0.15 | - | - | - | 1.94±0.21 | 0.29±0.05 | tr | 5.21±0.19 | 4.90±0.11 | 0.28±0.07 |
| **93** | (*E*)-β-Farnesene | sh | 1485 | 4.28±2.26 | 1.82±0.31 | 1.86±0.30 | - | - | - | - | - | - | 9.63±0.49 | 8.18±0.27 | 0.63±0.19 |
| **94** | cis-Muurola-4(14),5-diene | sh | 1460 | - | - | - | - | - | - | 0.15±0.00 | - | - | - | - | - |
| **95** | *allo*-aromadendrene | sh | 1461 | - | - | - | - | - | - | - | 0.14±0.00 | tr | - | - | - |
| **96** | Prococene I | chromene | 1462 | 3.04±1.73 | 0.34±0.07 | tr | - | - | - | - | - | - | - | - | - |
| **97** | γ-Muurolene | sh | 1477 | - | 0.06±0.00 | - | - | - | - | - | - | - | 0.15±0.01 | 0.05±0.00 | tr |
| **98** | Germacrene D | sh | 1480 | 13.40±4.59 | 10.86±0.14 | 9.35±0.74 | 0.16±0.02 | 1.99±0.01 | 0.41±0.11 | 0.07±0.00 | - | - | 27.03±0.44 | 23.55±0.31 | 3.33±1.33 |
| ***99*** | β-Selinene | sh | 1485 | - | - | - | - | - | - | 0.26±0.08 | - | - | - | - | - |
| **100** | Phenyl ethyl 3-methyl butanoate | nt | 1489 | - | - | - | - | - | - | 0.09±0.00 | - | - | - | - | - |
| **101** | *trans*-muurola-4(14),5-diene | sh | 1490 | - | 0.20±0.05 | - | - | - | - | - | - | - | - | - | - |
| **102** | *cis*-β-Guaiene | sh | 1493 | - | - | - | - | - | - | - | - | - | - | 0.18±0.00 | - |
| **103** | Valencene | sh | 1491 | - | - | - | - | - | - | - | - | - | - | 0.27±0.14 | - |
| **104** | Viriflorene | sh | 1493 | - | - | - | - | - | - | 1.66±0.29 | 0.73±0.03 | - | - | - | - |
| ***105*** | Bicyclogermacrene | sh | 1496 | 1.23±0.79 | 0.97±0.16 | 1.67±0.35 | 0.11±0.01 | 1.40±0.11 | 0.67±0,13 | - | - | - | 7.86±0.41 | 5.90±0.49 | 0.50±0.06 |
| ***106*** | Pentadecane | nt | 1500 | - | - | - | - | - | - | 0.07±0.00 | - | 0.90±0.05 | - | - | - |
| **107** | *trans*-β-guaiene | sh | 1501 | - | 0.13±0.00 | - | - | - | - | - | - | - | - | - | - |
| **108** | β-Bisabolene | sh | 1509 | 0.45±0.31 | 0.25±0.02 | 0.56±0.16 | - | - | - | - | - | - | - | - | - |
| **109** | *trans*-α-Cadinene | sh | 1513 | 0.10±0.00 | 0.14±0.00 | - | - | - | - | - | - | - | - | - | - |
| **110** | *trans*-γ-Cadinene | sh | 1514 | - | - | - | - | - | - | 0.27±0.10 | - | - | - | - | - |
| **111** | β-Curcumene | sh | 1517 | - | - | - | - | - | - | - | - | - | - | 0.51±0.03 | - |
| **112** | *cis*-Calamenene | sh | 1521 | - | - | - | - | - | 0.19±0.07 | - | 1.97±0.26 | 1.21±0.06 | - | - | - |
| **113** | β-Sesquiphellandrene | sh | 1524 | 0.16±0.00 | - | - | - | - | - | - | - | - | - | - | - |
| ***114*** | *trans*-Calamene | sh | 1532 | - | - | - | - | 0.36±0.15 | - | - | - | - | - | - | - |
| **115** | δ-Cadinene | sh | 1534 | - | - | - | - | - | - | 0.18±0.01 | - | - | 0.31±0.02 | 0.21±0.00 | - |
| **116** | α-Cadinene | sh | 1538 | - | - | - | - | - | - | - | - | - | tr | 0.20±0.00 | tr |
| **117** | Caryophellene oxide | os | 1581 | 0.08±0.00 | 0.21±0.08 | - | - | - | - | 0.07±0.00 | - | - | - | - | - |
| **118** | Prococene II | chromene | 1671 | 1.08±0.98 | 2.45±0.59 | 17.59±1.45 | - | - | - | - | - | - | - | - | - |
| **119** | Cadalene | sh | 1674 | - | 1.28±0.21 | - | - | - | - | - | 0.54±0.06 | - | - | - | - |
| **120** | *iso*-amyl dodecanoate | nt | 1846 | - | - | - | - | 1.30±0.20 | - | - | - | - | - | - | - |
| *Number of identified compounds (%>1%)* | |  |  | **29** | **40** | **26** | **16** | **24** | **24** | **31** | **32** | **23** | **37** | **40** | **42** |
|  | **Total Identified** |  |  | **99.71±0.08** | **99.81±0.13** | **98.64±0.93** | **96.95±0.44** | **96.57±1.38** | **97.19±0.61** | **99.63±0.00** | **100.0±0.00** | **99.67±0.35** | **98.15±1.51** | **98.02±0.23** | **99.47±0.53** |
|  |  |  |  | **Asteracea** | | | **Laminaceae** | | | | | | **Geraniaceae** | | |
|  |  |  |  | ***A. houstonianum*** | | | ***T. lemmonii*** | | | ***S. dorisiana*** | | | ***P. odoratissimum*** | | |
|  | **Class of Compounds** |  |  | **0** | **2** | **6** | **0** | **2** | **6** | **0** | **2** | **6** | **0** | **2** | **6** |
| Monoterpene hydrocarbons (mh) | | | | 33.90±13.78 | 52.77±1.64 | 44.45±0.78 | 63.32±0.08 | 20.39±1.56 | 16.97±10.62 | 14.86±0.41 | 64.03±3.05 | 43.63±0.33 | 6.91±1.50 | 9.25±0.34 | 76.17±0.52 |
| Oxygenated monoterpenes (om) | | | | 2.14±0.12 | 1.38±0.13 | 1.27±0.14 | 37.37±0.39 | 68.55±0.45 | 69.47±1.56 | 36.72±1.38 | 27.96±4.02 | 48.05±0.28 | 8.53±1.08 | 8.78±0.38 | 17.77±2.11 |
| **Total monoterpenes (TM)** | | | | **36.04±13.90** | **53.27±1.14** | **45.72±0.92** | **96.69±0.47** | **88.93±2.01** | **84.44±9.07** | **51.58±0.97** | **91.98±0.97** | **91.68±0.05** | **15.85±0.42** | **18.02±0.71** | **93.94±2.64** |
| Sesquiterpene hydrocarbons (sh) | | | | 54.36±13.49 | 41.93±1.03 | 35.07±0.52 | 0.26±0.03 | 6.34±0.43 | 2.76±0.33 | 45.39±0.66 | 8.29±0.72 | 7.99±0.40 | 80.71±1.90 | 76.20±1.46 | 7.58±0.85 |
| Oxygenated sesquiterpenes (os) | | | | 0.08±0.00 | 0.21±0.06 | - | - | - | - | 0.07±0.00 | - | - | - | - | - |
| **Total sesquiterpenes (TS)** | | | | **54.43±13.56** | **42.13±0.97** | **35.07±0.52** | **-** | **-** | **-** | **45.46±0.73** | **8.29±0.72** | **7.99±0.40** | **80.71±1.90** | **76.20±1.46** | **7.58±0.85** |
| Non-terpenoid derivatives (nt) | | | | 5.12±2.44 | 0.75±0.20 | 1.26±0.08 | - | 1.30±0.20 | - | 2.60±0.24 | - | - | 1.92±0.63 | 3.75±0.46 | 0.18±0.00 |
| Chromene | | | | 4.12±2.71 | 2.79±0.47 | 17.59±1.45 | - | - | - | - | - | - | - | - | - |
| Phenylpropanoid (pp) | | | | - | - | - | - | - | - | - | - | - | - | - | 0.72±0.01 |
